# Bronchopleural fistula following *M. abscessus* infection 11 years after lobectomy for lung cancer

**DOI:** 10.1186/2193-1801-2-568

**Published:** 2013-10-26

**Authors:** Mami Inayama, Tsutomu Shinohara, Mitsuteru Yoshida, Hiroyuki Hino, Nobuo Hatakeyama, Fumitaka Ogushi

**Affiliations:** Division of Pulmonary Medicine, National Hospital Organization National Kochi Hospital, 1-2-25 Asakuranishimachi, Kochi, 780-8077 Japan; Department of Clinical Investigation, National Hospital Organization National Kochi Hospital, 1-2-25 Asakuranishimachi, Kochi, 780-8077 Japan; Division of Thoracic Surgery, National Hospital Organization National Kochi Hospital, 1-2-25 Asakuranishimachi, Kochi, 780-8077 Japan

**Keywords:** Rapidly growing nontuberculous mycobacteria, Empyema, Airway destruction

## Abstract

Bronchopleural fistula (BPF) is a potentially fatal complication of lung cancer resection surgery that occurs during the healing process of the bronchial stump. However, the vulnerability of the healed surgical wound to overlapping acquired airway destruction has not yet been determined in detail. We herein present a case of fatal BPF following *Mycobacterium abscessus* (*M. abscessus*) infection, which occurred 11 years after right upper lobectomy for lung cancer. The findings of the present study suggest that patients with *M. abscessus* pulmonary disease in which airway destruction is progressing towards the bronchial stump of previous lobectomy should be considered for early completion pneumonectomy to prevent fatal BPF.

**Electronic supplementary material:**

The online version of this article (doi:10.1186/2193-1801-2-568) contains supplementary material, which is available to authorized users.

## Introduction

Bronchopleural fistula (BPF) is a rare, but potentially fatal complication of lung cancer resection surgery and has a high mortality rate (27.2 - 67 %). Management is very difficult once it has been diagnosed due to persistent empyema, refractory infection in the residual lungs, and subsequent sepsis (Lois and Noppen, 2005).

*Mycobacterium abscessus* (*M. abscessus*), a rapidly growing nontuberculous mycobacteria (NTM) that belongs to group IV of Runyon’s classification, has been well-known as a pathogen of skin and soft tissue infections following accidental trauma and surgery (Wallace et al., 1983; Chadha et al., 1998), and accounts for 80% of rapidly growing mycobacterial respiratory isolates. *M. abscessus* pulmonary diseases are rapidly progressive or multiresistant to antimicrobial drugs in many cases, and become intractable, which leads to severe airway destruction (Griffith et al., 2007).

We herein present a case of fatal BPF with empyema following *M. abscessus* infection, which occurred 11 years after right upper lobectomy for lung cancer.

## Case report

**Figure 1:**
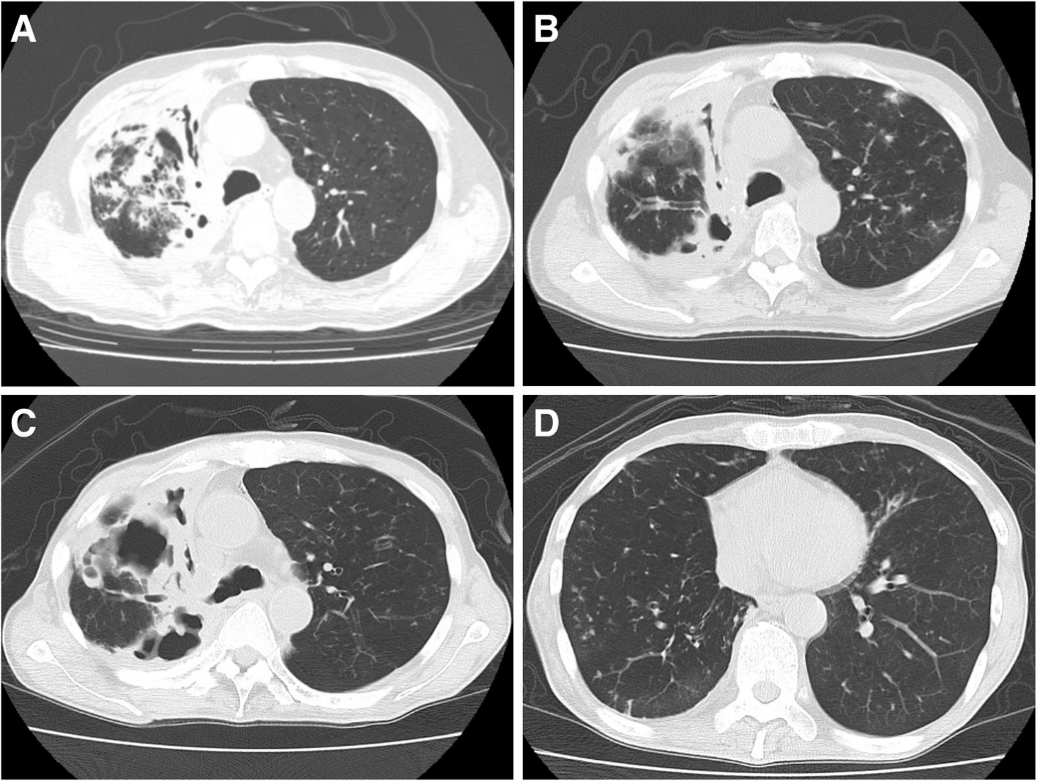
**Chest CT course before final admission. (A)** On first admission for the treatment of *M. intracellulare* pulmonary infection, showing a infiltrative shadow in the right upper lung field. **(B)** 1.5 years after *M. abscessus* was first detected in his sputum, revealing bronchiectasis and cavitary lesions in the middle lobe. **(C, D)** Six months before his final admission, showing bronchiectasis and cavitary lesions progressed towards the bronchial stump **(C)** and mild lesions in the right lower lobe **(D)**.

A 57-year-old male was diagnosed with primary lung cancer, and underwent right upper lobectomy. He was diagnosed with autoimmune hemolytic anemia at 62 years of age. Steroid therapy was initiated at 60 mg prednisolone/day, and this dose was subsequently reduced to 7.5 mg daily. He was referred to our hospital 3 months later for the treatment of *M. intracellulare* pulmonary infection in the right upper lung field. Chest computed tomography (CT) revealed an infiltrative shadow in the right upper lung field (middle lobe) (Figure 1A). He was administered clarithromycin and levofloxacin without discontinuance, and kanamycin for the initial 3 months. Cultures for NTM were subsequently negative. However, when the patient was 63 years of age, *M. abscessus* was identified in his sputum for the first time. Although treatments with imipenem/cilastatin and azithromycin were added intermittently, cavitary lesions and airway destruction in the right upper lung field gradually progressed towards the bronchial stump (Figure 1B and C). Lesions in the right lower lobe were mild at this time (Figure 1D). Since his disease was still localized to the right lung, we considered pneumonectomy. However, fearing a marked decline in cardiopulmonary function, the patient did not wish to undergo resection surgery.

**Figure 2:**
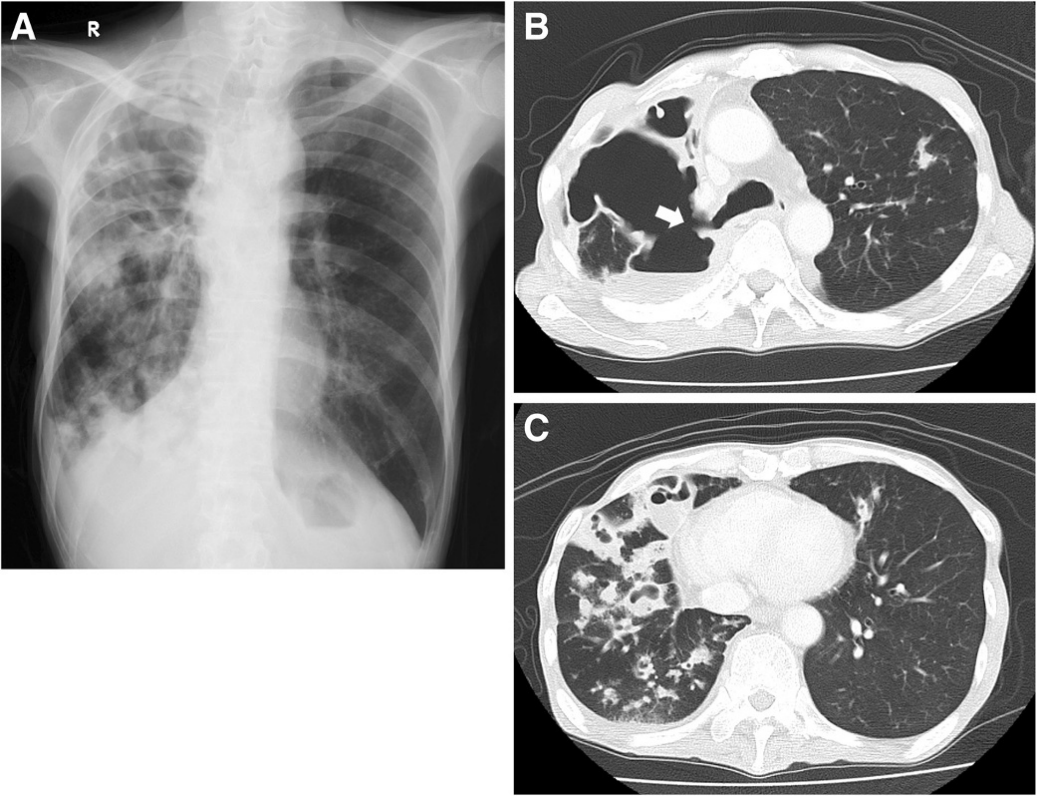
**Radiological examination on final admission. (A)** Chest radiograph showing an enlarged air space in the right upper lung field and a diffuse infiltrative shadow. **(B, C)** CT images demonstrating a large air space with niveau formation and multiple cavitary lesions in the residual lung, respectively. A right upper bronchial fistula (arrow) can be seen.

**Figure 3:**
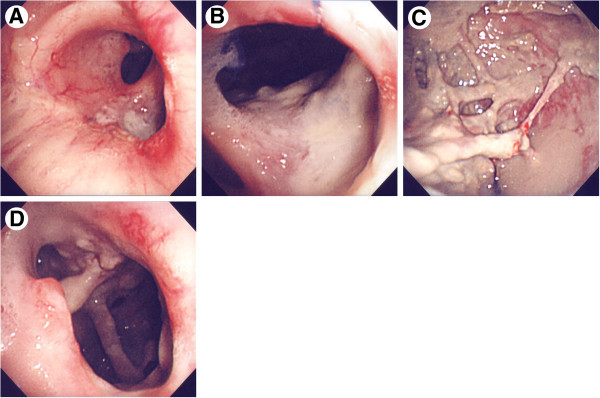
**Endoscopic images indicating massive airway destruction and empyema. (A)** Right main bronchus. **(B)** Fistula in the right upper lobe bronchus. **(C)** Right pleural cavity with irregularly adhered pleura and purulent matter. **(D)** Truncus intermedius.

At 67 years of age, he was admitted to our hospital with continuous fever and dyspnea. On admission, his height was 168 cm, weight 39 kg (BMI 13.8), and body temperature 38.1°C. Lung auscultation revealed decreased breath sounds in the right upper lung field and coarse crackles in the right lower zone. Laboratory data showed leukocytosis (WBC 11550/μl) and a high CRP level (10.26 mg/dl). Sputum examinations isolated Gaffky 5 of *M. abscessus,* but no other common bacteria. Chest X-ray showed an enlarged air space in the right upper lung field, and a diffuse infiltrative shadow in the right lower lung field (Figure 2A), which was not present 3 months earlier. Chest CT revealed a large space with air-fluid levels present (Figure 2B) and multiple cavitary lesions in the residual lung (Figure 2C). A right upper bronchial fistula was visualized on CT (Figure 2B). We speculated that *M. abscessus* infection extended to the bronchial stump, which was then gradually eroded by the uncontrollable mycobacterial infection, resulting in BPF with empyema, and that the disease state then triggered further progression of the infection to the residual lung. We performed an endoscopic examination and confirmed massive airway destruction in the right lung (Figure 3A, B and D) and fistula in the right upper lobe bronchus (Figure 3B), through which the pleural cavity with irregularly adherent pleura and purulent matter was directly observed (Figure 3C). *M. abscessus* was detected as a pathogen by bronchoscopic aspiration (equivalent to Gaffky 9). The patient was scheduled to undergo right pneumonectomy to control persistent infection. However, his respiratory condition deteriorated rapidly and he died before surgery could be performed.

## Discussion

Postoperative BPF can be classified into early and late BPF according to the time of occurrence: the former appears within a month of surgery, and the latter appears after more than one month (the average time of late BPF after lobectomy was reported to be 238.33 ± 164.12 days) (Jichen et al., 2009). Early BPF was shown to be related to perioperative technical problems, whereas late BPF was related to impaired healing of the bronchial stump (Varoli et al., 1998). Although several studies have identified some of the risk factors of postoperative early or late BPF, such as right pneumonectomy, infection, full-dose radiation, squamous carcinoma, and steroid use (Lois and Noppen, 2005; Jichen et al., 2009), few have focused on BPF following surgical wound healing.

On the other hand, non-postoperative BPF following necrotizing lung disease associated with chemotherapy or radiotherapy, persistent spontaneous pneumothorax, and lung necrosis complicating infection are less common (Lois and Noppen, 2005). Therefore, the vulnerability of the healed surgical wound to overlapping acquired airway destruction has not been fully determined. To the best of our knowledge, this is the first reported case of BPF after an extended period of time following lung cancer resection surgery, secondary to severe airway destruction caused by pulmonary NTM infection.

The frequency of NTM pulmonary disease is increasing, and is typically caused by slow-growing species such as *M. avium-intracellulare complex* (MAC) and *M. kansasii* in patients with or without preexisting lung disease (Griffith et al., 2007). However, as shown in our case, the microbial substitution of MAC to *M. abscessus* in NTM patients after long-term treatment with multiple drugs for previous MAC pulmonary disease has become a serious problem (Nei et al., 2007). In contrast with MAC infection, patients with *M. abscessus* infection require chemotherapy with parenteral antibiotics such as amikacin, cefoxitin, and imipenem for several weeks. However, this response is often temporary. *M. abscessus* pulmonary disease in our patient was also very difficult to eradicate and become refractory, leading to massive airway destruction (Figures 1, 2 and 3).

Surgical resection is commonly recommended for patients with localized *M. abscessus* pulmonary disease (Griffith et al., 2007). However, in many cases, lobectomy cannot fully remove bronchiectasis and damaged lung parenchyma due to an underlining pulmonary disease such as previous tuberculosis, MAC infection, and middle-lobe syndrome. In addition, the relatively high complication rate of pneumonectomy (Sherwood et al., 2005) was not suitable for the surgical treatment of our patient, who had a very low BMI, with a history of upper lobectomy. As a result, we missed the timing for surgery and BPF triggered the rapid progression of *M. abscessus* pulmonary disease, leading to respiratory failure.

The findings of our study suggest that patients with *M. abscessus* pulmonary disease in which airway destruction is progressing towards the bronchial stump of previous lobectomy should be considered for early completion pneumonectomy to prevent fatal BPF.

## Consent

Written informed consent was obtained from the patient’s family for the publication of this report and any accompanying images.

## Authors’ original submitted files for images

Below are the links to the authors’ original submitted files for images. Authors’ original file for figure 1 Authors’ original file for figure 2 Authors’ original file for figure 3
