# Supplementary figures and images for: Bronchopleural fistula following M. abscessus infection 11 years after lobectomy for lung cancer
Source: Springerplus. 2013 Oct 26;2(1):568. doi: 10.1186/2193-1801-2-568 (PMC3825059; doi:10.1186/2193-1801-2-568)

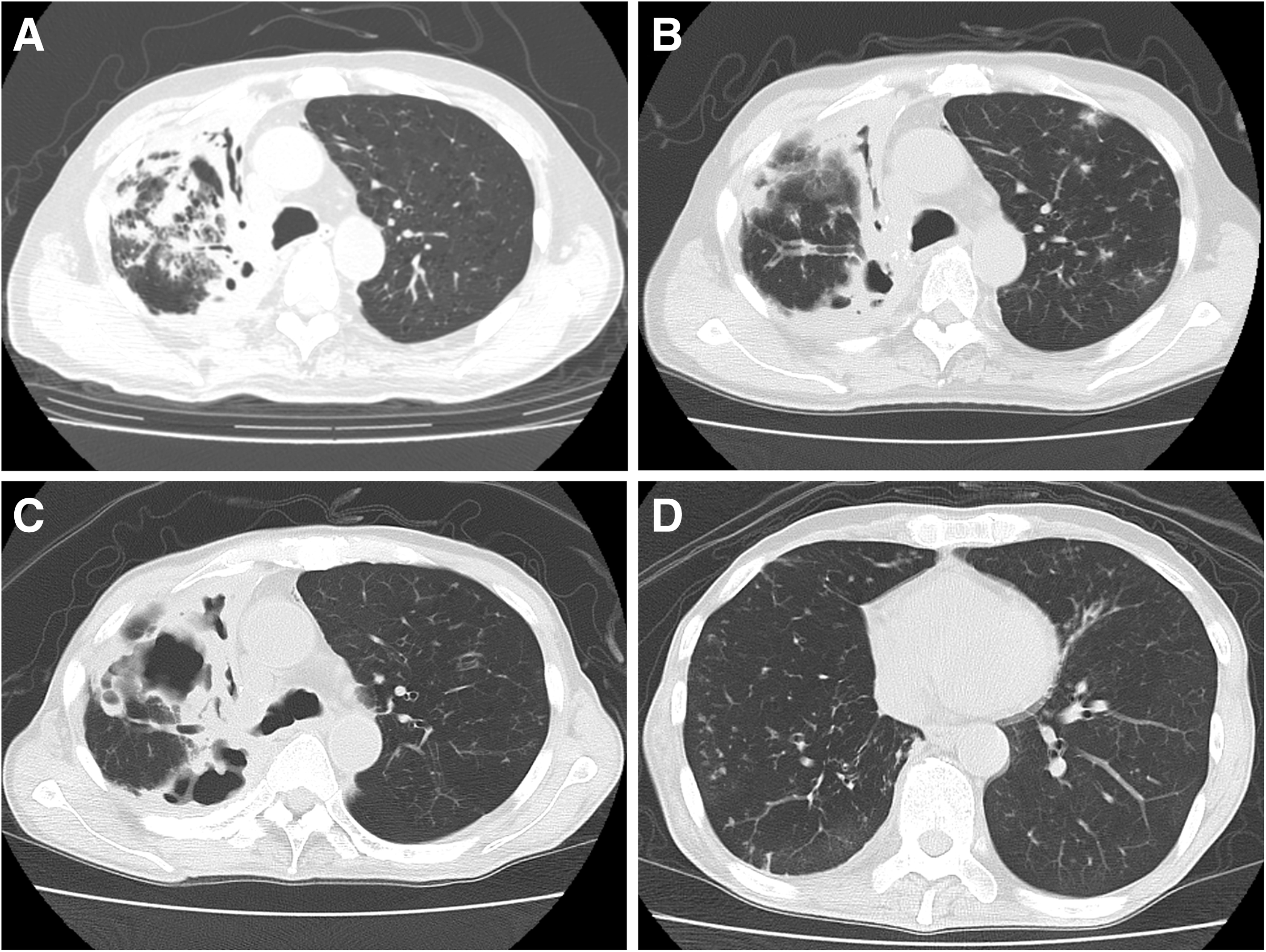

Supplement: Supplementary file 1 — Authors’ original file for figure 1 [file 40064_2013_624_MOESM1_ESM.tiff]

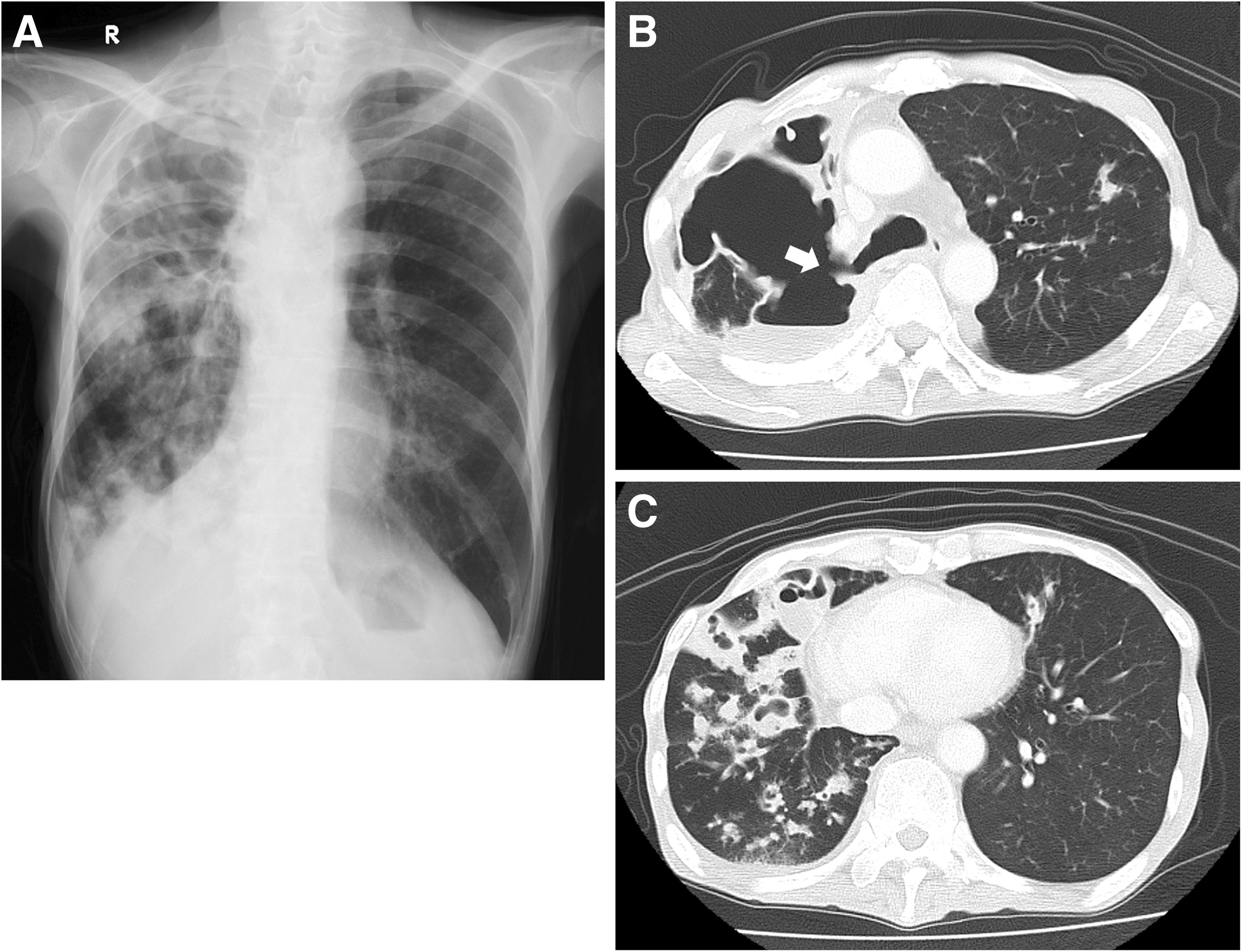

Supplement: Supplementary file 2 — Authors’ original file for figure 2 [file 40064_2013_624_MOESM2_ESM.tiff]

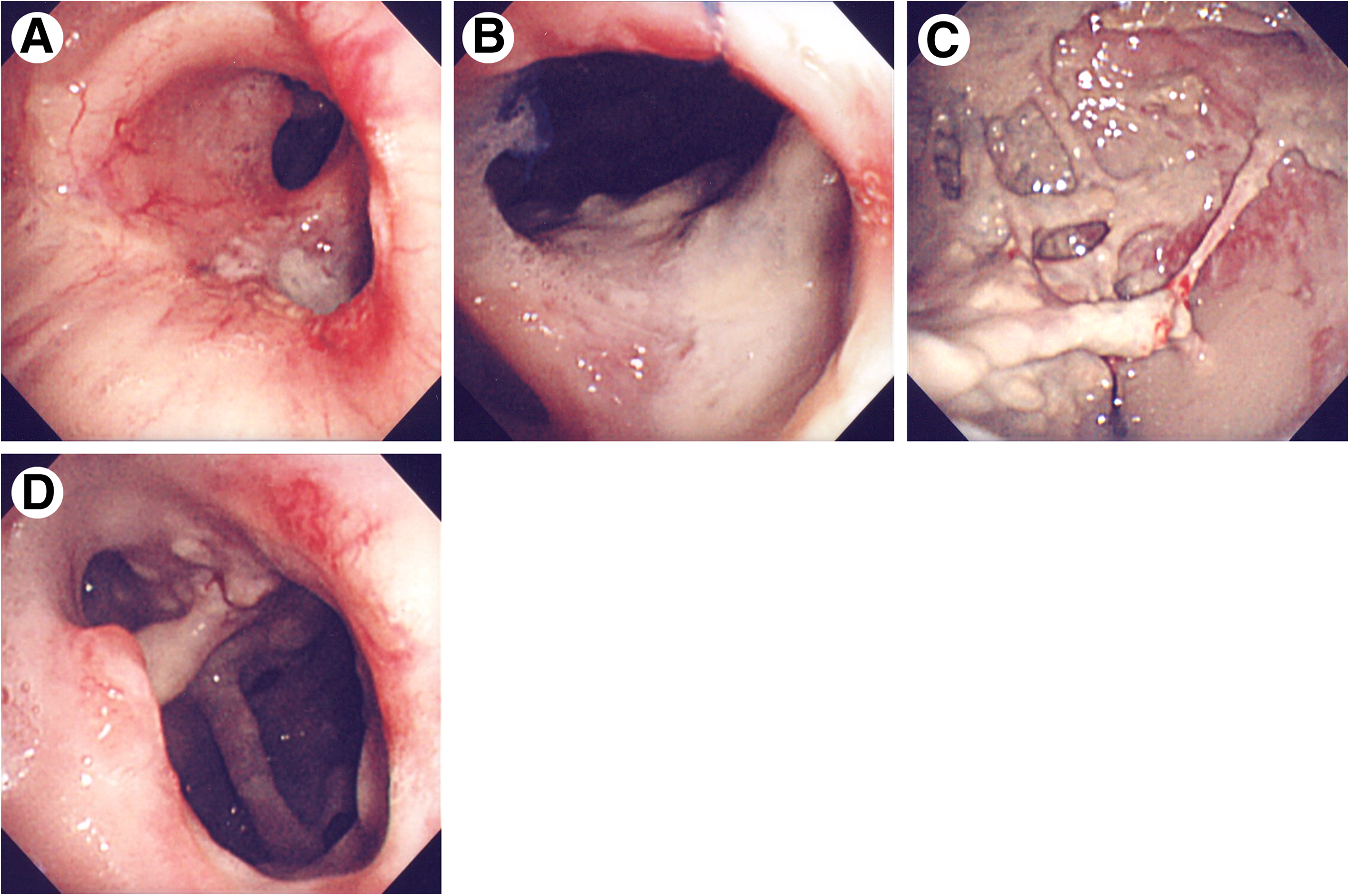

Supplement: Supplementary file 3 — Authors’ original file for figure 3 [file 40064_2013_624_MOESM3_ESM.tiff]
